# Quality of Life and Sexual Health in the Aging of PCa Survivors

**DOI:** 10.1155/2014/470592

**Published:** 2014-03-17

**Authors:** Mauro Gacci, Elisabetta Baldi, Lara Tamburrino, Beatrice Detti, Lorenzo Livi, Cosimo De Nunzio, Andrea Tubaro, Stavros Gravas, Marco Carini, Sergio Serni

**Affiliations:** ^1^Department of Urology, University of Florence, Careggi Hospital, Viale Gramsci 7, 50121 Florence, Italy; ^2^Department of Experimental and Clinical Biomedical Sciences, Section of Clinical Pathophysiology, University of Florence, Italy; ^3^Radiotherapy, University Hospital Careggi, University of Florence, Italy; ^4^Department of Urology, Sant'Andrea Hospital, University “La Sapienza”, Rome, Italy; ^5^Department of Urology, University Hospital of Larissa, Larissa, Greece

## Abstract

Prostate cancer (PCa) is the most common malignancy in elderly men. The progressive ageing of the world male population will further increase the need for tailored assessment and treatment of PCa patients. The determinant role of androgens and sexual hormones for PCa growth and progression has been established. However, several trials on androgens and PCa are recently focused on urinary continence, quality of life, and sexual function, suggesting a new point of view on the whole endocrinological aspect of PCa. During aging, metabolic syndrome, including diabetes, hypertension, dyslipidemia, and central obesity, can be associated with a chronic, low-grade inflammation of the prostate and with changes in the sex steroid pathways. These factors may affect both the carcinogenesis processes and treatment outcomes of PCa. Any treatment for PCa can have a long-lasting negative impact on quality of life and sexual health, which should be assessed by validated self-reported questionnaires. In particular, sexual health, urinary continence, and bowel function can be worsened after prostatectomy, radiotherapy, or hormone treatment, mostly in the elderly population. In the present review we summarized the current knowledge on the role of hormones, metabolic features, and primary treatments for PCa on the quality of life and sexual health of elderly Pca survivors.

## 1. Introduction

Prostate cancer (PCa) is the most common malignancy in elderly men. Age is a relevant risk factor, with a proven histological PCa being found in 60% of men by the age of 70 years and 80% by the age of 80 [1]. In fact, PCa is considered a chronic disease, needing a long period for initiation, development, and progression, through the development of early and later precancerous modifications, such as high-grade prostate intraepithelial neoplasia (HG-PIN), leading to the development of a clinically relevant cancer [2, 3]. Therefore, PCa is frequent in old men, likely becoming the prevalent cancer because of the ageing of population [4].

Although androgen receptor (AR) pathway is crucial for prostate cancer growth and progression, evidence supporting a favorable risk-benefit ratio of androgen deprivation therapy (ADT) is currently limited to high-risk PCa or metastatic disease [5, 6]. Furthermore, hypogonadism is common in elderly men and men who have PCa: the symptoms of hypogonadism, such as depression, erectile dysfunction (ED), and lower urinary tract symptoms, can impair a man's quality of life (QoL) [7]. Therefore, androgens and AR play a critical role in management of elderly men with PCa.

The current literature suggests an association between metabolic syndrome (MetS) and PCa, although the evidence for a causal relationship remains unknown [8]. In particular, a recent review pointed out that men with MetS seem to have more likely high-grade and advanced PCa: moreover, they resulted in greater risk of progression and cancer specific death, even if the overall analyses did not reveal any association between MetS and the risk to develop the disease [8]. Therefore, MetS should be assessed as a new domain in basic and clinical research in elderly men with PCa.

The primary goal of any definitive treatment of PCa is the improvement of survival and QoL: although surgery, radiotherapy, and hormone therapy can lead to long-term survival, these treatments can cause lasting side effects [9]. Therefore, patients survival has to be considered in treatment decision making, but patients' quality of life must also be considered before and after any treatment [10]. Moreover, an accurate assessment of QoL in PCa patients must be performed with validated, self-reported, and disease specific instruments [11]. Therefore, there is a need for a tailored approach in the management of PCa in the elderly men, to avoid unnecessary intervention with permanent adverse event [12].

The aim of present review is to summarize the current knowledge on the role of androgens pathways, metabolic factors, and primary treatments on the overall QoL and sexual health of elderly PCa survivors.

## 2. Endocrinological Aspects of Prostate Cancer

### 2.1. The Role of Androgens and of Androgen Receptor (AR) in Carcinogenesis and Progression of Prostate Cancer

Prostate volume and function are age- and androgen-dependent [13] and in hypogonadal subjects therapy with testosterone restores the volume of the prostate to that of eugonadal men [14]. Androgens and AR play a fundamental role in the development of PCa which is androgen-dependent for its growth, as demonstrated in the pioneering work of Huggins and Hodges [15] who showed that castration causes complete regression of the disease.

How actions of AR become tumorigenic and lead to uncontrolled growth remains poorly understood. In a high percentage of PCa, fusions between the androgen-dependent gene TMPRSS2 and ETS transcription factors (such as ERG) occur through chromosomal translocations [16], leading to elevated expression of these oncogenic factors under androgen control. However, whether TMPRSS2:ETS fusions are sufficient to promote PCa is discussed [17, 18] and the initial enthusiasm about such chromosomal aberrations has been dampened by the controversial results of clinical studies investigating their role in PCa progression [19].

**Figure 1:**
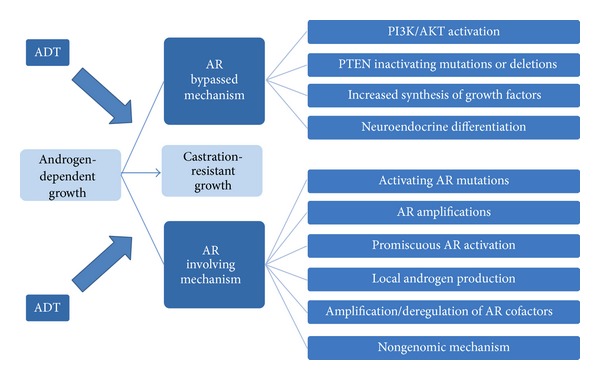
Schematic representation of the main pathways involved in development of castration-resistant prostate cancer (CRPC). ADT: androgen-deprivation therapy, AR: androgen receptor. Modified from 43.

Androgen deprivation therapy (ADT) represents a valuable treatment of metastatic PCa. However, ADT provides palliation but not cure and most PCa regrow as castration-resistant PCa (CRPCa) able to survive and grow in a milieu virtually deprived of androgens. The detailed mechanisms of why ADT ultimately fails and a more aggressive cancer recurs remain unclear (Figure 1). In the past decade, based on in vitro or in vivo evidence, several hypotheses involving the AR have been generated to explain development of CRPCa, such as AR mutations (found in about 20% of metastatic specimens) or amplifications that confer the ability to bind other steroids and even antiandrogens (acting as agonists), changes in AR-coregulators interactions, and activation of AR by growth factors or other signal pathways (reviewed in [20]). In addition, recent work has highlighted the role of intraprostatic androgen synthesis as the driving force of recurrent disease (see below for further details).

Interestingly, low expression of mutated AR may drive in vitro growth of CRPCa cell lines also by nongenomic (rapid signalling) mechanisms [21]. However, more studies are needed in order to better understand the role, if any, of nongenomic AR signalling in PCa growth and progression. These AR-involving hypotheses do not completely explain why patients receiving ADT tend to have an earlier development of more aggressive cancer. Alternative pathways of growth and invasion may develop in PCa cells (Figure 1) bypassing the necessity of androgens: among these, PTEN inactivating mutation has been found in a high proportion of PCa [22] leading to suppression of apoptotic pathways and consequent uncontrolled growth. Neuroendocrine differentiation also plays an important role in development of CRPCa [23]. In summary, development of CRPCa is a very complex event, potentially involving both androgen-regulated and androgen-alternative pathways (Figure 1). Such a complexity makes the development of therapeutic strategies very difficult, and, as today, CRPCa is basically incurable.

Currently, research is mainly directed to understand the role of these multiple pathways and their interregulation with the aim of identifying potential therapeutical targets. One hot topic of research is aimed at understating the role of AR. In a recent survey of the literature concerning the relationship between AR expression in PCa specimen and disease prognosis, we have highlighted the conflicting results reported so far [24]. These studies evidenced both the highly variable expression of AR among different cancers and a different relation with prognosis. Most studies did not find any association between AR expression and prognosis, including a large one by Minner et al. [25], whereas some studies found an association between high AR expression and better or worse prognosis. Although such contrasting findings may depend on several factors [24], one possible explanation may be related to a different role of AR depending on its location (stroma or epithelium) in the tumor. Recently, a mouse cancer model lacking the AR only in the prostatic epithelium and/or stroma has been generated (ARKO-TRAMP) [26, 27]. These mice paradoxically develop poorly differentiated PCa and, most importantly, restoration of AR function in epithelial basal cells leads to tumor suppression. Conversely, restoration of AR in stromal cells stimulates cancer progression, supporting a differential role of AR in PCa depending on its location. Studies in mice models suggest that stromal AR may promote prostate tumorigenesis via induction of proinflammatory cytokines/chemokines expression [28].

These results substantiate in vitro studies showing that enforced expression of AR in AR-negative PCa cells decreases the metastatic/invasive potential of the cells [26, 29–35]. In a recent paper evaluating the role of androgen signalling in epithelial-mesenchimal transition, Zhu and Kyprianou [36] demonstrated that overexpression of AR in PCa cell lines suppresses androgen-induced epithelial-mesenchimal transition, suggesting that downregulation of AR occurring in androgen-deprived condition [37] may facilitate mesenchimal transition and promote metastasis [36]. There is also evidence that inducing AR expression in PCa cells by targeting methylation of promoter increases differentiation of carcinoma cells and suppresses self-renewal/proliferation of stem cells and tumorigenesis [38].

Clinical data supporting a differentiating role of AR in PCa and, as such, limiting invasiveness have been also published. Following androgen ablation metastatic PCa is promoted in vitro [39] and there is clinical evidence that intermittent ADT benefits patients in PCa progression [40]. In addition, patients with CRPCa displaying amplification of AR gene survive longer than patients without amplification [41]. Results of long-term survival in the PCa prevention trial with the 5 alpha-reductase inhibitor finasteride demonstrated that, although the risk of developing PC is decreased, the Gleason score of developing cancers is significantly higher in the finasteride group and, overall, no difference in life expectancy between the treated and placebo group was observed [42]. Finally, there is evidence in the literature that, in some instances, CRPCa may benefit from androgen-replacement therapies [43–45]. Overall, these studies suggest that AR may have both negative and positive roles in PCa progression by regulating cell growth and invasion ability [46, 47].

In such a complex scenario, it is clear that more studies are needed to define the role of AR in the different PCa compartments. In addition, investigations should be aimed at evaluating the different AR variants present in the tumors. Indeed a recent study [48], performed in a small series of CRPCa bone metastases (*n* = 30), demonstrated that expression of AR variants lacking the ligand binding domain was associated with poor prognosis and shorter survival rates. If these results will be confirmed in a larger series of subjects, they can open new therapeutic perspectives to target other portions of AR. Of interest, an AR antagonist, EPI-001, able to bind the N-terminal domain of AR, has been recently developed [49, 50]. This antagonist has been found to reduce the growth of CRPCa xenografts [51].

### 2.2. Prostate Cancer in Hypogonadal Men

Although a causative role for circulating androgens on PCa has been envisaged since the Huggins and Hodges studies, data clearly showed that such a link is at best unproved. In a meta-analysis of 18 prospective studies, including almost 4000 men with incident PCa and 6500 control subjects, no associations were found between the risk of PCa and serum concentrations of Testosterone (T), calculated FT, DHT, and other androgens [52]. Furthermore, some authors have documented that low serum T is associated with more aggressive, ADT-resistant tumors suggesting that low levels of androgens create a selective pressure for PCa cells leading to androgen-independence ([53, 54]; for review see [55, 56]).

In line with these data, the pooled odds ratio for Testosterone Replacement Therapy (TRT) derived from 19 randomized clinical trials was 1.09 (0.48–2.49, 95% CI) for PC and 1.19 (0.67–2.09, 95% CI) for PSA > 4 ng/dL or 1.5% increase during study (for review see [55, 56]). Based on critical analysis of clinical trials and on the aforementioned experimental data on PCa cell lines, so far 11 investigators evaluated the effect of TRT even in PCa patients, with the aim of inducing differentiation in the tumor [55, 56]. Overall these studies included 279 subjects previously treated with radical prostatectomy or radiotherapy. In the vast majority of patients no association with progression or clinical recurrence was reported. Despite this evidence, it should be recognized that the number of reported cases is still small and heterogeneous. In the absence of randomized controlled trials (RCT), the concept of using TRT for PCa survivors is debatable. Accordingly, current recommendations suggest limiting TRT to symptomatic hypogonadal men successfully treated for PCa, after a prudent interval, although the length of that interval is not specified [57].

### 2.3. Intraprostatic Synthesis of Steroids: Role in PCa Progression

As mentioned above, intraprostatic androgen synthesis may support PCa cell growth even in the virtual absence of androgens contributing to development of CRPCa [13]. There is evidence that androgen levels may remain elevated in the prostate during ADT [58, 59]. Moreover, androgens have been found in locally recurrent CRPCa [60] and in distant metastasis [61]. These studies suggest that the PCa may acquire the capability to synthesize androgens although a direct proof can be only obtained by demonstrating the occurrence of steroidogenic machinery in the cells. Results of the latter experiments are contrasting [62, 63]. In particular, in a recent study [62], expression of the steroidogenic enzymes CYP17A1 and HSD3B1, essential for androgen synthesis, has been detected at low levels only in 19 of the 88 tumor samples leading to the conclusion that intratumoral steroid biosynthesis has a limited contribution. However, the elevated expression of 5 alpha-reductase found in CRPCa samples [63] suggests that de novo steroidogenesis may occur bypassing the requirement of T by 5 alpha-reduction of adrenal precursor steroids [13]. Targeting androgen synthesis in CRPC with abiraterone acetate, a potent inhibitor of CYP17, resulted to be safe and well tolerated, leading to a reduction of the risk of death and increased median survival of some months compared to placebo [64]. At present ongoing investigations are evaluating the efficiency of abiraterone acetate (in combination with other treatments) in the early stages of PC. Efforts are currently directed to understand the mechanisms of resistance to abiraterone acetate and how to prevent it.

### 2.4. Modifications of Sex Hormone after Radical Treatment for Prostate Cancer

Several studies have analyzed the modifications in the levels of T and gonadotropins following radical treatment, producing controversial results [65–68]. In particular, in 55 males treated with radical prostatectomy (RP), a remarkable increase in T, luteinizing hormone (LH), and follicle-stimulating hormone (FSH) has been reported 1 year after RP [69]. These data were confirmed by Olsson et al. [66]. In a group of 49 men, LH and FSH were increased by 71 and 63%, respectively, 12 months after RP without any evident changes in T, suggesting that the hypothalamic pituitary axis was inhibited in patients with PCa and that this inhibition has been removed following RP [69].

Recently, we enrolled 100 men affected by PCa in a single center prospective study, with the aim to evaluate the changes in the serum levels of T, LH, and FSH within the first 3 months after RP for clinically localized PCa and to analyze the correlation between LH and T at various follow-up times [67]. As expected, we found a remarkable positive correlation between T and LH before surgery (*r* = 0.370; *P* < 0.0001), but not 1 month after RP (*r* = 0.109; *P* = 0.303). Three months after prostatectomy, the correlation between T and LH was restored (*r* = 0.273; *P* = 0.054). Therefore, our data demonstrated that RP can induce an early significant decline in the T levels and a compensatory increase in LH and FSH levels. These data have a critical relevance, suggesting that hormone modifications could have an important role in the loss and the subsequent recovery of both urinary continence and potency [70]. Three months after RP, the full recovery of T levels, with persistent high levels of gonadotropins, seems to delineate the features of compensated hypergonadotropic hypogonadism.

To confirm these data and to analyze the influence of T on sexual activity and urinary continence in men with PCa, we consecutively enrolled 257 patients treated with RP in our center [71]. As expected both age and BMI have a negative impact on preoperative T levels. Moreover, in men with normal T, urinary continence was significantly correlated with sexual function and sexual bother (*r* = 0.2544: *P* = 0.01 and *r* = 0.2512: *P* = 0.01), whereas this correlation was lost in hypogonadal men.

## 3. Metabolic Syndrome and Prostate Cancer in Elderly Men

Metabolic syndrome describes the combination or clustering of several metabolic abnormalities including central obesity, dyslipidemia, hypertension, insulin resistance with compensatory hyperinsulinemia, and glucose intolerance [72–74]. Recently, epidemiological, histopathological, molecular pathological, and clinical studies have provided emerging evidence of a possible role of MetS and its components in PCa development and progression [74, 75].

Although the only well-established risk factors associated with PCa are age, race, and family history, the large geographical variations in PCa risk suggest that lifestyle and environmental factors may also contribute to its etiology. The possibility to prevent and treat MetS and its components led to novel therapeutic approaches that have been proposed as a new frontier in the prevention and treatment of PCa [74, 76, 77].

### 3.1. Definition, Epidemiology, and Pathophysiology

MetS is a constellation of physiological and biochemical abnormalities characterized by diabetes or high fasting glucose, central obesity, abnormal cholesterol and triglyceride levels, and hypertension [78]. Currently, the two most widely used definitions are those proposed by the National Cholesterol Educational Program Adult Treatment Panel III (NCEP:ATP III) and by the International Diabetes Federation (IDF) focusing on abdominal obesity measured by waist circumference. In contrast, the World Health Organization (WHO) and the European Group for the study on Insulin Resistance (EGIR) definitions are principally focused on IR [73, 74].

Prevalence of MetS increases linearly from the age of 20 until age of 50, when it plateaus and affects more than 40% of the population in the United States and nearly 30% in Europe [79, 80]. Similar to western countries, the prevalence of MetS is rapidly increasing in developing countries, ranging from 9.8% in males from urban north India, to 16.3% in Morocco, to 25.4% in urban Brazil, to 33.5% in South Africa, and to 33.7% in Iran [74, 78, 79]. People with MetS are estimated to have twice the risk of developing cardiovascular disease compared to healthy individuals and a fivefold increased risk of type-2 diabetes. MetS has been recently linked to a number of urological diseases including PCa [72, 75]. Although IR and obesity are considered at the core of the pathophysiology of MetS, a number of other factors can also be involved in its pathogenesis and potential interactions [73, 74].

In most cases MetS develops as a result of poor eating habits and/or sedentary lifestyles which are associated with IR and obesity. IR occurs when there is a decrease in the responsiveness of peripheral tissues (skeletal muscle, fat, and liver) to the effect of insulin with a concomitant hyperinsulinemia [80]. Hyperinsulinemia is also responsible for stimulating Insulin Growth Factor-1 (IGF) production in the liver. IGF-1 is a potent mitogenic factor and apoptosis inhibitor which has been linked with PCa risk [81].

**Figure 2:**
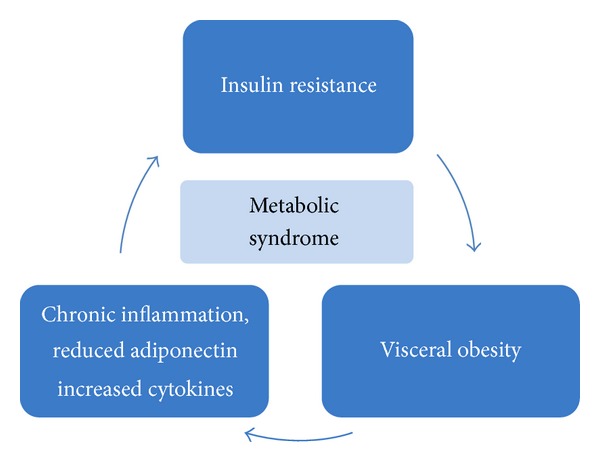
The pathophysiological loop of metabolic syndrome.

Central obesity is also considered an early step in the development and progression of MetS. Visceral adipose tissue secretes various bioactive substances known as adipocytokines which can induce IR and have proinflammatory and proatherogenic effects (Figure 2). Cytokines including resistin, leptin, tumor necrosis factor alpha (TNF-*α*), interleukin-6 (IL-6), C-reactive protein (PCR), fibrinogen, and plasminogen activator inhibitor (PAI-1) are normally increased in obese patients and in patients with DMT2. On the contrary, adiponectin, is lower in individuals with visceral fat accumulation. Adiponectin stimulates glucose metabolism and fatty acid oxidation in the muscle, enhances insulin sensitivity in the liver, increases free fatty acid oxidation, reduces hepatic glucose output, and inhibits monocyte adhesion and macrophage transformation to foam cells within the vascular wall [80, 82]. Visceral adiposity may also contribute to hypogonadism, frequently associated with MetS in men, through increased aromatase activity. Its increased activity in obese patients raises estradiol levels, which results in feedback inhibition at the level of the hypothalamus/pituitary to lower T leading to hypogonadrotropic hypogonadism. Elevated estrogen levels lead to a further increase in visceral adipose deposition creating a self-sustained loop [74, 80, 82].

MetS has also been associated with a state of chronic, low-grade inflammation. Several studies showed that patients with MetS were more likely than those without to have elevated levels of a marker of inflammation such as C-reactive protein (CRP) as well as proinflammatory cytokines such as TNF-*α*, IL-8, IL-6, and IL-1*β* [83, 84].

### 3.2. Relationship between MetS and PCa

MetS has frequently been associated in human and animal models with carcinogenesis (see Table 1) [85]. It has recently been suggested that evaluating MetS as a single condition may be an inappropriate approach to investigating PCa risk. Specifically, combining all the multiple components of the syndrome into a single variable may confound or obscure the independent effects and interactions of these metabolic components on PCa risk [86]. Each of the primary components of the MetS have been individually observed to be directly associated with PCa risk. DMT2 has been associated with a reduction in PCa risk, probably in relation to the changing action of insulin over the course of diabetes progression [86, 87]. The presence of hypertension may increase PCa risk, in part through increased sympathetic nervous system activity, which can result in androgen-mediated stimulation of PCa cell growth [86]. Men with lower plasma cholesterol were less likely to develop high-grade PCa than men with higher concentrations; this effect might be mediated by several pathways including androgen metabolism and intracellular cholesterol-mediated signaling [88, 89]. Most recent large studies suggest that obesity is associated with a decreased risk of low-grade disease, but an increased risk of high-grade and advanced PCa [74].

Moreover, obesity was associated with an increased risk of intraoperative and perioperative complications and with a worse functional outcome, in men treated with RP [90]. In particular, obese men are at threefold greater risk of intraoperative complications and blood transfusions than not-obese men (adjusted odds ratio (OR) = 3.116, *P* < 0.001, and OR = 2.763, *P* < 0.050, resp.). Furthermore, the risk of needing at least two pads per day is two and a half times greater in men with a waist circumference of at least 102 cm than in those with a WC below 102 cm (adjusted OR = 2.435, *P* = 0.007).

In conclusion, further basic and clinical studies are needed to evaluate this association by investigating all these metabolic conditions as a whole and to better evaluate the role the MetS and its mediators with the development and progression of PCa.

## 4. Measurement of Quality of Life and Sexual Health in Men with Prostate Cancer

Any treatment of PCa can affect urinary and sexual activity, psychosocial function, and overall wellbeing. Several validated questionnaires have been used to asses QoL after RP. An effective evaluation should consider at least 3 categories of QoL: (1) the organ specific function (urinary and sexual); (2) the physical status and the mental health in the patients with any type of cancer; (3) the general health status. The following validated questionnaires are the most accurate to assess the overall health and quality of life for men with PCa.

### 4.1. UCLA-PCI

The University of California-Los Angeles Prostate Cancer Index (UCLA-PCI) is very accurate to evaluate all aspects related to QoL before and after any treatment for PCA. This questionnaire investigates urinary, bowel and sexual function (UF, BF, and SF), and bowel and sexual bother (UB, BB, and SB) and has been designed either for urologists or radiotherapists [97, 98]. The majority of questions were assigned a score from 0 to 100 (0 = worse health; 100 = better health).

### 4.2. European Organisation for Research and Treatment of Cancer (Cancer Generic): EORTC QLQ-C-30

This validated questionnaire, designed to evaluate the QoL in men affected by or treated for any cancer, is a 30-item questionnaire composed of multi-item scales and single items that reflect the multidimensionality of the quality-of-life construct [99]. It incorporates five functional scales (physical, role, cognitive, emotional, and social), three symptom scales (fatigue, pain, and nausea/vomiting), and a global health and QoL scale.

### 4.3. Short Form-12 (SF-12)

The SF-12 is the short version of the SF-36 questionnaire [100]. Through 12 questions, it allows investigating, instead of the 8 original scales, only two indices: the Physical Component Summary (PCS) and the Mental Component Summary (MCS). The strengths of this form are the brevity and relative ease of use from both patients and physicians. For every question there are from 3 to 5 options.

### 4.4. International Index of Erectile Function (IIEF)

The IIEF questionnaire is a validated multidimensional self-administered questionnaire used to assess the erectile function and the response to treatment in clinical trials. A score of 0–5 is awarded to each question that evaluates 4 domains of sexual health: sexual desire, erectile function, orgasmic function, and intercourse satisfaction [101]. Recently, a short form (IIEF-5), based on 5 questions instead of the original 15 questions, has been used.

## 5. Impact of Primary Treatment for Pca on QoL and Sexual Health

Currently, more than half of all PCa are clinically localized at the diagnosis, with a 5-year biochemical disease-free survival above 85% [102]. Treatment options for clinically localized PCa include watchful waiting, radical prostatectomy, radical external beam radiation, and hormone treatment. Nevertheless, while active surveillance may have a minimal impact on either QoL or sexual health, more invasive therapies can lead to clinically significant and lasting side effects [103].

In particular, erectile dysfunction and urinary incontinence after RP, bowel, urinary, and sexual complications after radiotherapy or sexual and continence and mood bothersome after hormone treatment can have a negative impact on all the aspects of QoL, including vitality, social and physical, and emotional limitations.

### 5.1. Primary Treatment in the Elderly Men

The appropriate management of the older men can be a challenge: elderly men are more likely to be diagnosed with higher-grade cancer and the presence of comorbidity and functional decline can impact on treatment tolerance and side effects [104, 105]. Therefore, RT and/or ADT are more commonly used because they are less invasive and do not carry the risk of surgery and anesthesia.

PC is often diagnosed as a result of routine screening in asymptomatic patients, so the development of late treatment sequelae may be particularly alarming [106], also considering the improvement of life expectancy in elderly population (over 17 years at 65 years old) [107].

QoL is a key criterion in the choice of treatment, particularly for early PC or elderly patients, but it is difficult to assess despite the availability of validated questionnaires [108–112]. Late QoL impact is well documented in the literature, but most of the longest QoL studies did not include pretreatment evaluation [113, 114] or collected it retrospectively [115–117].

Generally, recent cohort prospective studies of patients show a different pattern of late sequelae: an increased prevalence of urinary incontinence after prostatectomy, of urinary irritative obstructive symptoms after brachytherapy, of bowel side effects after EBRT, whereas sexual dysfunction as common late event after all these treatment including ADT [110, 111, 118–120].

In particular, erectile dysfunction (ED) is a critical point related to QoL in men treated for PCa and it is strongly associated with depression and significant distress [119, 121, 122]. ED in these patients is the result of several factors: anatomic changes after surgery or radiotherapy, not only hormonal therapy but also psychological and social factors and finally specific comorbidities that often occur in elderly men (metabolic syndrome, diabetes, obesity, osteoporosis, reduced muscle mass, and strength) [123–126].

### 5.2. Radical Prostatectomy

The major concerns for patients undergoing RP are postprostatectomy incontinence (PPI) and ED. Both QoL and sexual health after RP are strongly dependent on patient age, aging, tumor characteristics, and disease progression [112]. While slight urinary of sexual dysfunction can lead to important bother in younger men, remarkable symptoms can generate minimal bother in the elderly. QoL and sexual health can progressively change owing to anatomical modifications, treatment for PCa, or the natural aging [127]. Therefore, age at time of treatment and perspectives in both sexual and general health have the same clinical relevance of tumor features [128]. Finally, tumor progression or recurrence after RP can generate anxiety and fear that can further worsen QoL and sexual health [129]. In a retrospective, cross-sectional study, enrolling 595 men with PCa treated with radical treatment as primary therapy, we demonstrated that pretreatment tumor characteristics (clinical stage, bioptical Gleason score, and total PSA), treatment timing (age at time of treatment, follow-up duration after treatment, and age at time of follow-up), and posttreatment outcomes (biochemical recurrence and hormonal status) had a remarkable impact on QoL.

One of the leading determinants of QoL and sexual health after RP is the surgical approach: a more conservative procedure, sparing neurovascular bundles, bladder neck, and proximal urethra can strongly increase the chance of better functional outcomes [130, 131]. The decision for the surgical approach is usually a compromise between patient's desire to preserve sexual activity and the eligibility to a conservative surgery based on tumor characteristics (PSA, Clinical stage, bioptical Gleason Score). In a prospective survey on 2,408 PCa patients treated with RP, we demonstrated that at least 737 men (30.6%) were interested in preservation of sexual activity, but not eligible for a nerve-sparing procedure, based on their high-risk PCa features [132]. For 372 (50.5%) of these patients a nerve sparing approach (monolateral or bilateral) was chosen: in these highly selected cases, surgeons' strategy was performed in accordance with patients' desire, without compromising surgical margin status.

The complete recovery of urinary continence after RP is mandatory to preserve general health and to maximize the outcomes of sexual rehabilitation: after catheter removal, most patients reported some level of urinary incontinence [132]. In a multicenter prospective study, we enrolled 1972 men with full continence preoperatively and complete postoperative data: 1 month after RP, 644 (32.7%) were fully continent, 810 (41.1%) were using 0-1 pad/day, and 518 (26.3%) >1 pad/day. Age and nerve sparing were not significant predictors of continence recovery after RP, while preoperative erectile function allowed predicting PPI: the integrity of pelvic vasculature and nerves prior to RP was determinant to avoid of early PPI.

ED and urinary continence can improve even beyond more than 1 year postoperatively, with an average time to sexual and urinary recovery of >6 months [133]. Moreover, Phosphodiesterase type 5 inhibitors (PDE5-I), either in nightly or on-demand dosing, are the gold standard to recover sexual function after nerve-sparing prostatectomy [134]. In a multicenter RCT we have randomized men treated with nerve sparing prostatectomy for localized PCa into 3 groups: (1) PDE5-Is on demand; (2) PDE5-Is once a day; (3) placebo [135]. PDE5-IS improved continence recovery compared with placebo (Improvement of Urinary Function at 3, 6, and 9 months after PDE5-Is once a day versus placebo: *P* = 0.042, *P* = 0.044, and *P* = 0.039, resp.): the positive effect of PDE5-I on continence recovery, even in the absence of the prostatic gland, suggested a direct activity of PD5-Is on lower urinary tract by a pathway not including prostate. Therefore, the long-term use of PDE5-Is after RP can strongly influence the general QoL, the urinary function, and the sexual health.

**Figure 3:**
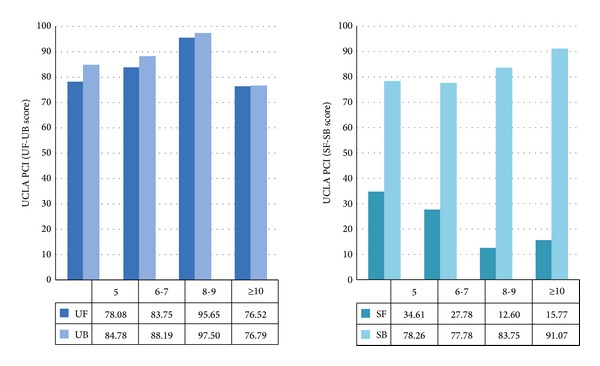
Comparison of function and bother in long-term disease-free survivors after nerve sparing RP without hormone treatment: UF: urinary function; UB: urinary bother; SF: sexual function; SB: sexual bother; RP: radical prostatectomy (adapted from [70]).

During the natural aging process in disease-free survivors after RP, both urinary and sexual symptoms and bother can be strongly modified, due to hormonal modification and both vascular and nerve impairment. Moreover, after long-term disease-free follow-up, several men reconsider their QoL status [136]. In two tertiary referral center for PCa we recruited 367 men treated with RP (for clinically localized PCa), without biochemical failure (PSA ≤ 0.2 ng/mL) at the follow-up ≥5 years, with the aim to evaluate long-term general QoL and sexual health in elderly PCa survivors [70]. Older men presented worse urinary continence regardless of age at time of surgery or follow-up duration. Moreover, after more than 8 years after nerve sparing RP without hormone treatment, patients reported substantial sexual dysfunction, but, interestingly, they were minimally sexually bothered (see Figure 3). Our data confirmed that slight urinary incontinence is poorly tolerated even after several years of complete cancer control, while sexual dysfunction is better tolerated, in the daily life of long-term disease-free survivors, perhaps because patients consider ED as a part of their natural aging.

### 5.3. Radiotherapy

Current indications for external-beam radiotherapy (EBRT) in PC include primary treatment (in localized intracapsular tumors or combined with ADT in locally advanced and high-risk PC), adjuvant treatment in patient with adverse pathologic features, (extracapsular extension, positive surgical margins), and salvage radiotherapy (after radical prostatectomy) [137].

However, over recent years, radiotherapy (RT) has seen major advances such as the introduction of intensity-modulated radiation therapy (IMRT) and image-guided RT (IGRT) [138, 139]. The higher radiation doses that can be delivered to the prostate by these new techniques, whilst sparing surrounding organs, have improved progression-free survival and reduced acute and late toxicities [140, 141]. Several studies investigated this aspect of QoL [119] with a short (1–3 years) or intermediate (4-5 years) follow-up, while longer-term outcomes remain largely unknown. Regarding age of patients, some studies have found equal rates of both acute and late side effects in all age groups [142, 143] while others have found older age to be associated with faster onset and more frequent side effects [71]. A prospective trial evaluating patients more than one year following EBRT treatments with final dose of 70–72 Gy found that older age and diabetes were predictive of both preexisting ED and post-EBRT acquired ED [144].

Sanda et al. concluded a substantial decline from baseline of sexual function at 2 years after surgery, but only a moderate decline after EBRT or brachytherapy. Recovery of sexual function was worse in patients treated with androgen suppression combined with radiotherapy, in older patients, obese patients, and patients with a larger prostate size and a high pretreatment PSA. The patient's QoL concerning sexual function was also significantly related to satisfaction in the partner [111].

Pardo et al., in a Spanish study, had similar results after a follow-up of 3 years in patients treated by surgery or EBRT or brachytherapy [145], as well as Rice et al. in a USA study: the authors concluded that EBRT had no significant impact on sexual function at 12 months and may be offered to older patients with minimal QoL impact [146]. As reported in the studies of Potosky et al. [115], the Prostate Cancer Outcomes Study (PCOS), and the one of Miller et al. [113], the patients treated by surgery had an improvement in their sexual function at 2 years after diagnosis, whereas the patients treated by EBRT had slight declines: a possible explanation of this result is the older age of the group of patients treated by radiotherapy. Instead in the Litwin's et al. report long-term sexual function scores were better among surgical patients, but return to baseline was more rapid in patients treated by EBRT [147].

In their report of a long-term follow-up, Resnick et al. [148] described that, although patients undergoing RP were more likely to have ED at 2–5 years, at 15 years the prevalence of ED is very common, affecting 87% of men treated by surgery and 93.9% of men treated by EBRT: it is matter of debate if this decline is due to late sequelae of oncologic treatments, to normal aging process, or to a combination of these factors. Van der Wielen et al. studied the correlation between ED and dose to penile bulb in patients treated with doses of 68–78 Gy: but there was no relation found [149]. Mangar et al. demonstrated that a dose received by 90% of the penile bulb (D90) >50 Gy was significantly associated with ED (*P* = 0.006) [150], results comparable to the outcomes of the study of Wernicke et al. [151].

The Radiation Therapy Oncology Group 9406 trial examined 158 men with a regular erectile function at baseline and found a greater risk of impotence with a penile mean dose >52.5 Gy (*P* = 0.039) [152]. So these data are suggestive for a correlation between dose to penile bulb and ED, but more prospective studies are indispensable for predicting preservation of sexual function.

Regarding alternative fractionations, the low *α*/*β* ratio of PC causes the high sensitivity of these cells to higher doses for fraction than other tumors [153, 154], even if it is well known that hypofractionated treatments can result in increased rate of late toxicity. In a Canadian prospective study, at 39 months of follow-up, moderate and severe distress related to urinary and bowel symptoms was minimal (3% and 5% of patients, resp.), and the rate of sexual dysfunction was in line with the studies with conventional fractionations [155].

Finally, the few studies available concerning QoL after dose-escalated radiotherapy (thanks to advances in radiotherapy techniques) suggest an increased radiation dose does not result in decline of QoL [156–159].

### 5.4. Androgen Deprivation Therapy (ADT)

Approximately 50% of men with PCa receive ADT at some time after diagnosis, and most will take it for at least 2 to 3 years [160, 161]. Currently, luteinizing hormone-releasing hormone (LHRH) agonists are the most frequently used agents for ADT. However, other agents including high-dose estrogen, high-dose ketoconazole, abiraterone, and LHRH antagonists can also be used to achieve a castrate level of T. Single-agent antiandrogen therapy is also used as a form of ADT, but it is more likely to reach lower serum T levels [162].

ADT presents several symptoms of “castration syndrome” as side effects, based on low serum T concentration. The symptoms include loss of libido and sexual interest, erectile dysfunction, general fatigue, decreased intellectual ability, depression, loss of muscle strength, increased abdominal fat mass, and loss of vigor [163]. Several cross-sectional studies have described the effect of ADT on self-reported physical function; these studies consistently found that ADT treated men reported decreased physical function in comparison to nontreated [164, 165].

Regarding the side effects of hormonal therapy, in a Canadian retrospective study of Joly et al. [166, 167] patients treated with ADT for at least 3 months for localized PCa both as adjuvant therapy and as biochemical relapse were enrolled. Tests were administered to assess the: patients had significantly poorer scores than controls, especially for urinary disorders and sexuality (*P* < 0.01). The urinary and sexual symptoms may be primarily due to the prior local treatment. ADT contributed to deterioration in sexual functions, but this study was not designed to address this question; of the patients, 90% reported sexual problem, in agreement with results of other studies [168]. However elderly patients often report that urinary symptoms have a greater impact than sexual functions on global QoL [166, 169].

In an Australian longitudinal study the authors investigated the change to QoL and T level in men starting an intermittent maximal androgen blockade program. Two hundred and fifty men were recruited in this multicentre study: T suppression leads to a significant reduction in global QoL and deterioration in most function as sexual function. Complete loss of libido increased from 37.2% before treatment to 72.2% after hormonal deprivation. Complete sexual inactivity increased from 54.3% to 86.9%. Following treatment cessation, T recovery was gradual and median time to eugonadal levels of the hormone was 9.3 months with an improvement in emotional function, sexual function, fatigue, sleep, and hot flushes [170].

In the American study of Lubeck, QoL of 1178 newly diagnosed patients was examined (mean age at diagnosis was 73 years) which were enrolled in the Cancer of the Prostate Strategic Urologic Research Endeavor Database. General and disease specific QoL outcomes were measured with tests at study entry and quarterly thereafter. Patients were randomized in 3 groups: ADT, surveillance, radical prostatectomy, or EBRT. Men receiving ADT reported poorer urinary and sexual function and a higher rate of urinary and sexual symptoms than patients selecting surveillance. ADT and surveillance QoL scores remained low in the year after treatment, whereas men treated by RP showed improvement in these scales [167].

In the Australian phase 3 trial of Denham, all patients were given six months of leuprorelin, and radiotherapy to the prostate and seminal vesicles after 5 months from randomisation. After leuprorelin, patients were given either no further treatment or an additional 12 months of leuprorelin. In addition to androgen suppression, men who were randomly allocated to the two bisphosphonate groups were given zoledronic acid for 18 months. In this study, 18 months of androgen suppression worsened the adverse changes in the “patients-reported-outcome” score caused by 6 months of androgen suppression and radiotherapy. However, these increases were restricted to only sexual activity, hormone treatment related symptoms, fatigue, and financial problems at 18 months after randomization. The increases were also restricted in time [171].

Mature survival data from men with previously untreated, locally-advanced disease reveal that bicalutamide monotherapy provides survival benefits that do not differ significantly from castration, while offering important advantages with respect to the maintenance of physical capacity and sexual interest [172]. Also the study of Stav confirms that sexual interest appears to be better preserved with bicalutamide than with castration [173].

In the two largest phase III studies comparing bicalutamide 150 mg/die monotherapy with castration (orchiectomy or the LH-RH agonist goserelin acetate) in 1453 patients, the combined analysis at 12 months showed that bicalutamide was associated with a significant advantage for sexual interest compared with castration (*P* = 0.029), although a decrease was recorded in both groups [174].

## 6. Conclusions

In conclusion, the present review underlines the double role of androgens and the androgen receptor in the development and proliferation of PCa as well as in maintaining a correct functional state of the prostate of elderly men. Evidence in the literature suggests that maintaining a correct function of the androgen receptor may limit PCa progression by keeping a more differentiate state of the cells. Although more RCT are needed to better define the risk/benefit of androgen therapy in elderly men previously cured for PCa, current evidence indicates that treatment with androgens of hypogonadal men with previous PCa may be safe and may ameliorate both sexual health and QoL.

There are several lines of evidence regarding the emerging role of MetS and its components in PCa development and progression. Moreover, MetS can be associated with a state of chronic, low-grade inflammation, in particular in elderly men. We have summarized the evidence about the involvement of MetS in the pathogenesis of PCa, particularly of high-grade disease and we suggested that MetS should be assessed as a new domain in basic and clinical research in elderly men with PCa. In particular, all the components of MetS should be adequately assessed either before or after any treatment of PCa.

It is mandatory to use validated questionnaire to provide adequate details to patients not only regarding urinary and bowel symptoms, but also regarding their sexual function in order to avoid anxiety in patients and their families, to provide adequate medical and psychological counseling, and to analyze the progressive modifications during the follow-up of PCa survivors.

The adverse effects of surgery, radiotherapy, or androgen deprivation may be more pronounced in the elderly population, especially those with lower functional status and increased comorbidities, in particular regarding their sexual health. Therefore it is important to consider the specific benefits and risks for each treatment modality as they apply to the elderly because of the greater risk in both short- and long-term postoperative complications and mortality following any radical treatment.

## Figures and Tables

**Table 1:** Relevant clinical studies of the relationship between MetS and prostate cancer.

Authors, year	Study design	Country	Population	Time	Age years (range or mean ± SD)	Cohort size	Exposure assessment: MetS criteria	Number of cases	Results (outcome: PCa)	Level of evidence
Laukkanen et al., 2004 [91]	Longitudinal cohort study	Finland	Kuopio communities	1984–2001	42–62	1880 (White)	WHO	56	Risk increase (RR: 1.94, 95% CI: 1.06–3.53)	2b
Håheim et al., 2006 [92]	Longitudinal cohort study	Norway	Oslo study	1972–1998	40–49	15 933 (White)	Upper quartile levels ATP III criteria	507	Risk increase (RR: 1.56; 95 %CI: 1.21–2)	2b
Martin et al., 2009 [93]	Longitudinal cohort study	Norway	Nord-Trondelag Health study (HUNT 2)	1996–2005	48 ± 16.4	29 364 (White)	NCEP: ATP III	687	No association (HR: 0.91, 95% CI, 0.877–1.09)	2b
Beebe-Dimmer et al., 2009 [94]	Case-control study	USA	Gene Environment and Prostate Cancer study (GECAP)	2001–2004	62 ± 10.4	881 (56% White; 44% African-American)	NCEP: ATP III	637	Risk increase in African-American population (OR: 1.71, 95% CI; 0.97–3.01)	3
Tande et al., 2006 [95]	Longitudinal cohort study	USA	Atherosclerosis Risk in Communities (ARIC);	1987–2000	45–64	6429 (49% White; 61% African-American)	NCEP: ATP III	385	Risk reduction (RR: 0.77; 95% CI, 0.51–1.05)	2b
Kheterpal et al., 2012 [96]	Longitudinal cohort study	USA	Robotic radical prostatectomy	2005–2008	45–65	2756	BMI ≥30 and ≥2 of the following: hypertension, diabetes or elevated blood glucose, and dyslipidemia	357	Greater pathology Gleason grade (≥7: 78% versus 64%, *P* < 0.001) and pathologic stage (≥T3 disease: 43% versus 32%, *P* < 0.001)	3
C. De Nunzio et al., 2011 [78]	Cohort study	Italy	Prostate biopsy cohort study	2009-2010	47–83	195 (White)	NCEP: ATP III	102	No association (OR: 0.97, 95% CI: 0.48–1.95); Increased risk for Gleason score ≥7 in pts with PCA (OR: 3.82, 95% CI: 1.33–10.9)	3
