# Corrigendum to “Quality of Life and Sexual Health in the Aging of PCa Survivors”

**DOI:** 10.1155/2015/675101

**Published:** 2015-02-11

**Authors:** Mauro Gacci, Elisabetta Baldi, Lara Tamburrino, Beatrice Detti, Lorenzo Livi, Cosimo De Nunzio, Andrea Tubaro, Stavros Gravas, Marco Carini, Sergio Serni

**Affiliations:** ^1^Department of Urology, University of Florence, Careggi Hospital, Viale Gramsci 7, 50121 Florence, Italy; ^2^Department of Experimental and Clinical Biomedical Sciences, Section of Clinical Pathophysiology, University of Florence, Italy; ^3^Radiotherapy, University Hospital Careggi, University of Florence, Italy; ^4^Department of Urology, Sant'Andrea Hospital, University “La Sapienza”, Rome, Italy; ^5^Department of Urology, University Hospital of Larissa, Larissa, Greece

In the paper “Quality of Life and Sexual Health in the Aging of PCa Survivors” there are 2 mistakes at page 9, in the paragraph “5.4. Androgen Deprivation Therapy (ADT)”: “[162]” has to be replaced by the words “with LHRH agonists,” while “[162]” has to be shifted at the end of the second and third sentences (lines 6 and 9, resp.).

